# Immediate effects of photobiomodulation on maximum tongue pressure: a randomized clinical study

**DOI:** 10.1590/2317-1782/e20240139en

**Published:** 2025-02-21

**Authors:** Ester Florens Guerra Gouvêa, Lorena Moreira Marra, Vanessa Mouffron Novaes Alves, Mariana Rodrigues Batista, Andréa Rodrigues Motta, Renata Maria Moreira Moraes Furlan

**Affiliations:** 1 Graduação em Fonoaudiologia, Universidade Federal de Minas Gerais – UFMG - Belo Horizonte (MG), Brasil.; 2 Programa de Pós-graduação em Ciências Fonoaudiológicas, Universidade Federal de Minas Gerais – UFMG - Belo Horizonte (MG), Brasil.; 3 Departamento de Fonoaudiologia, Universidade Federal de Minas Gerais – UFMG - Belo Horizonte (MG), Brasil.

**Keywords:** Low-Level Light Therapy, Muscle Strength, Speech, Language and Hearing Sciences, Stomatognathic System, Tongue

## Abstract

**Purpose:**

To verify the immediate effects of infrared laser photobiomodulation on maximum tongue pressure.

**Methods:**

This is a randomized clinical study with 72 healthy adults of both sexes, with a mean age of 24.6 years, standard deviation of 4.6, no craniofacial anomalies, no signs or symptoms of temporomandibular disorder, no contraindications to phototherapy, and who did not continuously use muscle relaxant or anti-inflammatory medications. Participants with lingual frenulum changes were excluded from the sample. Maximum tongue pressure was measured using the Iowa Oral Performance Instrument (IOPI) before and after irradiating low-level laser at a wavelength of 808 nm on three points on the anterior portion and three on the posterior portion of the tongue. Participants were randomly allocated into four groups of 18 individuals each: G4, irradiated with 4 J per point; G7, irradiated with 7 J per point; CG, which did not receive irradiation; and PG, subjected to the same procedures as G4 and G7, but without laser activation – i.e., without irradiation.

**Results:**

no statistically significant differences were found between the maximum anterior and posterior tongue pressures when comparing pre- and post-intervention values. Although without statistical significance, the mean values increased slightly in the groups that received irradiation and decreased in the non-irradiated groups.

**Conclusion:**

no differences were found between the maximum anterior and posterior tongue pressures when comparing the pre- and post-intervention pressure values.

## INTRODUCTION

The tongue is a muscular organ belonging to the stomatognathic system, acting in nutrition and communication functions^(1)^. It has intrinsic and extrinsic muscles, with fibers in different directions (vertical, longitudinal, and transverse), giving it a wide range of movement possibilities^(2)^. The intrinsic muscles originate and are inserted in the tongue itself and are responsible for its changes in shape, while the extrinsic muscles originate in adjacent structures and act mainly in its movement in all directions^(3)^.

Tongue pressure can be defined as the capacity of this structure to exert force in a given area^(4)^. The scientific literature constantly evaluated this variable, as some clinical conditions are associated with decreased tongue strength – e.g., mouth breathing^(5)^, obstructive sleep apnea^(6)^, and primary snoring^(6)^. Individuals with changes in this strength may have impaired functions of the stomatognathic system^(7)^.

When a decrease in tongue strength is detected, the muscle rehabilitation process generally consists of myotherapy and myofunctional therapy^(1)^. Myotherapy is based on muscle exercises, while myofunctional therapy uses assisted functional training to rehabilitate the structures and functions of the stomatognathic system^(8)^.

Photobiomodulation, specifically low-level laser, is one of the adjuvant resources that has been currently used in myotherapy to accelerate the process of muscle strength recovery^(9)^. Laser has a biomodulatory action in the body – i.e., it acts on tissues by stimulating or inhibiting the chemical and/or physiological cell functions. Thus, photobiomodulation can improve muscle performance, reduce fatigue, increase strength, and relax the muscles^(10)^. These effects are justified by the action of light on cell mitochondria, specifically in cellular respiration, intensifying the production of adenosine triphosphate (ATP), which provides energy for muscle contraction^(11)^. Although the photobiomodulation efficacy to increase strength in some skeletal muscle groups has been proven^(9,12,13)^, few studies have addressed orofacial muscles^(14,15)^.

A study with women aged 19 to 43 years compared the immediate effects of photobiomodulation on electromyographic fatigue, using laser at wavelengths of 660 and 808 nanometers (nm) on the orbicularis oris muscle. They applied 4 J of energy to two points on the upper lip and two on the lower lip, totaling 8 J per segment. However, no significant differences were found in the measures before and after irradiation^(14)^. Another study, with a sample of men and women aged 18 to 33 years, compared the difference in lip pressure before and after applying laser at three different radiation intensities (1 J, 4 J, and 7 J) to six points on the orbicularis oris muscle. It found that the 7 J dose improved the performance of the orbicularis oris muscle, increasing the maximum pressure^(15)^.

No study has been identified to date investigating the effect of photobiomodulation on the tongue muscles – thus, there is no scientific evidence to validate photobiomodulation for this muscle or indicate which dose is most effective. Given the above, the general objective of this study was to investigate the immediate effects of photobiomodulation on tongue pressure, with the initial hypothesis that low-level irradiation at infrared wavelengths would be capable of increasing the maximum pressure of the tongue muscles.

## METHODS

This randomized, double-blind clinical trial was carried out after approval by the Research Ethics Committee of the Federal University of Minas Gerais (UFMG), under CAAE number 17622619.0.0000.5149 and opinion 3.581.053. All participants signed an informed consent form. The research was registered on the REBEC clinical trials platform under number RBR 7p58r6.

The study was conducted at the Observatory of Speech-Language-Hearing Functional Health at the UFMG Medical School. The non-probabilistic sample had 72 adults – 54 female and 18 male students and/or employees of the institution, aged 19 to 40 years (mean = 24.6, standard deviation = 4.6). The inclusion criteria were the absence of craniofacial anomaly, signs and/or symptoms of temporomandibular disorder, and contraindication to phototherapy; and no continuous use of muscle relaxants and/or anti-inflammatory medications. The following were considered contraindications for photobiomodulation: pregnancy, glaucoma, undiagnosed lesion on or near the area to be irradiated, infection at the application site, and history of cancer. Participants with lingual frenulum changes, diagnosed through the Tongue Frenulum Evaluation Protocol^(16)^, were excluded from the sample.

Data were collected in three stages – initial assessment, application of low-level laser, and reassessment.

### Stage 1 – Initial assessment

Participants were initially interviewed to verify the eligibility criteria (absence of craniofacial anomaly, signs and/or symptoms of temporomandibular disorder, pregnancy, glaucoma, injury in or near the area to be irradiated, infection at the application site, history of cancer, and continuous use of muscle relaxants and/or anti-inflammatory drugs) and evaluate the lingual frenulum, using the Tongue Frenulum Evaluation Protocol^(16)^. Next, the maximum tongue pressure was evaluated using the Iowa Oral Performance Instrument (IOPI). This device has an air bulb connected to a pressure transducer, measuring maximum pressure and muscle resistance. The air bulb is 3.5 cm long, 1.0 cm in diameter, and connected to an 11.5 cm plastic tube. As this bulb is pressed, the device captures pressure changes, providing values ​​in kPa on the device's screen. The IOPI is the gold standard instrument to objectively assess tongue pressure and resistance^(17)^.

Participants were instructed to remain seated in a chair with 90º flexion between hips, knees, and ankles and an upright posture, guided by the Frankfurt Plane, during tongue pressure measurements. These were made by positioning the bulb first on the anterior portion and then on the posterior portion of the tongue.

The bulb was positioned in the central part of the tongue, behind the central incisors, to measure the maximum anterior pressure. As for the maximum posterior pressure, the bulb was positioned with its anterior limit parallel to the beginning of the first molars^(18)^. The same instruction was given to all participants, who were asked to press the bulb against the palate with the tongue, with the maximum force possible, for approximately 2 seconds. Three measures were taken in each position (anterior and posterior of the tongue), with 30-second intervals between them^(18)^. The mean of the three pressure values ​​obtained in each position was considered for analysis.

### Stage 2: Applying low-level laser

Photobiomodulation was performed with low-level laser, using DMC^®^ equipment, Therapy EC model (São Paulo, Brazil). The irradiation parameters are described in Chart 1.

**Chart 1:** Photobiomodulation parameters

Irradiation parameters	Values in Groups G4 and G7
Wavelength	808 nm (infrared)
Operation mode	Continuous
Output power	100 mW
Output spot diameter	3.54 mm
Output spot area	0.09842 cm^2^
Energy per point	4 J and 7 J
Energy density (flux) per point	40.64 J/cm^2^ (G4) and 71.12 J/cm^2^ (G7)
Application time per point	40 s (G4) and 70 s (G7)
Number of points	6
Total irradiation time	4 min (G4) and 7 min (G7)
Total energy	24 J (G4) and 42 J (G7)
Application mode	Stationary mode with contact

The 72 research participants were randomly allocated by drawing lots into four groups of 18 participants each. Each group and the energy radiated are described in detail below:

- Group 1 (G4): Comprising 14 females and four males, with a mean age of 25.5, SD of 5.9, minimum of 20, and maximum of 36 years. They were irradiated with 4 J per point, with a total energy of 24 J and a total irradiation time of 4 minutes.
- Group 2 (G7): Comprising 14 females and four males, with a mean age of 24.6, SD of 4.0, a minimum of 20, and a maximum of 33 years. They were irradiated with 7 J per point, with a total energy of 42 J and a total irradiation time of 7 minutes.
- Control Group (CG): Comprising 15 females and three males, with a mean age of 24.6, SD of 5.0, a minimum of 19, and a maximum of 40 years. They were not irradiated.
  ● Placebo Group (PG): Comprising 10 females and eight males, with a mean age of 23.8, SD of 3.6, minimum of 19, and maximum of 31 years. They underwent the same procedures as G4 and G7, with the same application time and total irradiation time (4 or 7 minutes, defined randomly per participant), but the device was not activated.

**Figure 1:**
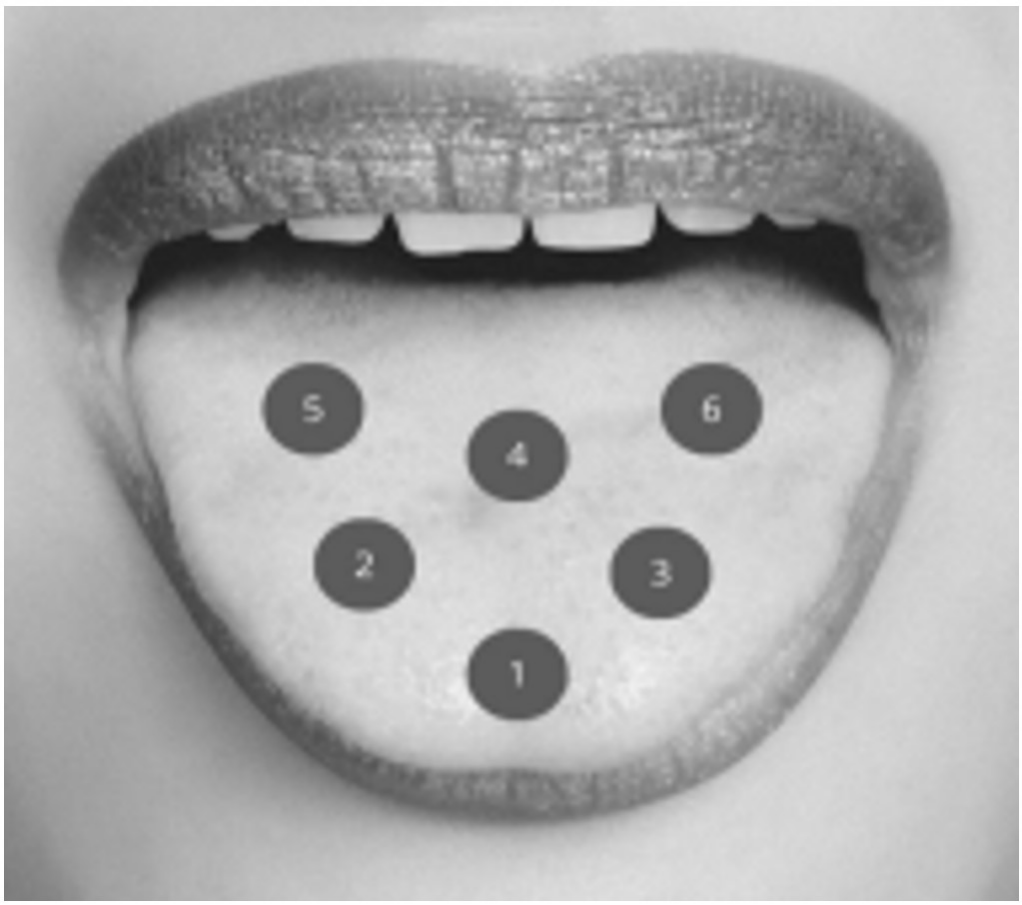
Laser application points on the tongue surface

Source: Pexels^(19)^ (Adapted by the author)

Point contact irradiation was applied in all groups, at six points on the tongue – three points in the anterior third and three points in the posterior third, with 1 cm between them, as shown in Figure 1^(19)^.

**Figure 2:**
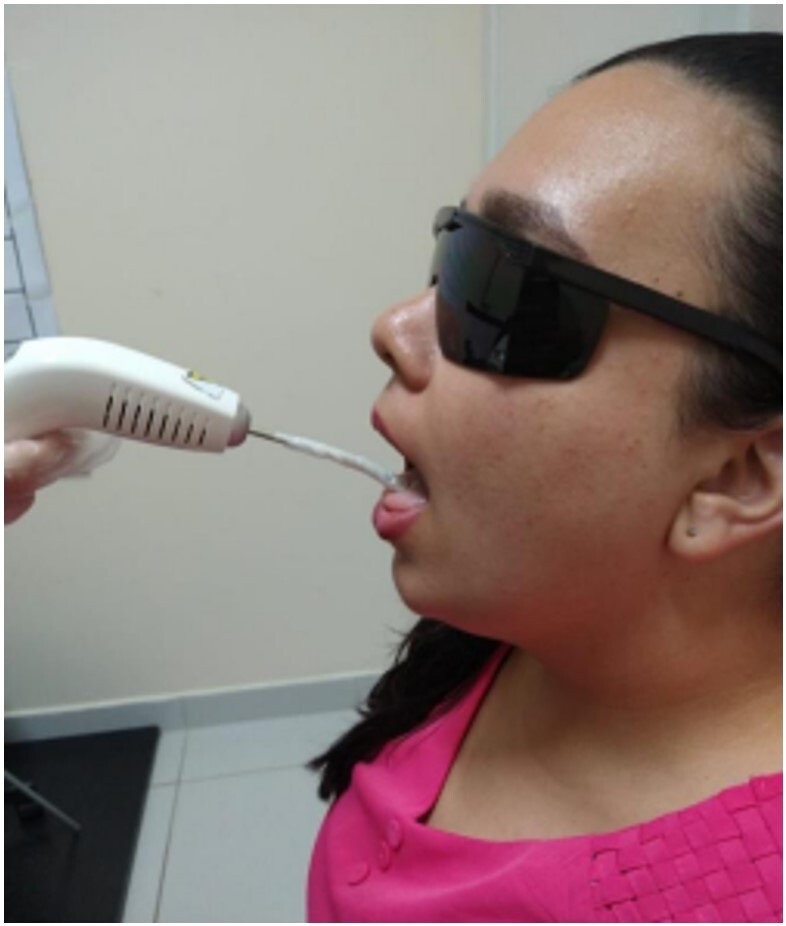
Laser application positioning

Source: Author’s files (2023)

It was necessary to prevent the irradiation procedure from causing tongue muscle fatigue. The anterior tongue points were irradiated first, uninterruptedly, followed by the posterior points. Participants were also instructed to open their mouths during photobiomodulation and keep their tongues comfortably on the floor, as shown in Figure 2.

The irradiation was performed by placing the tip of the equipment on the participant's tongue mucosa. The device was sanitized with 70% alcohol before each application, also using disposable plastic film to cover the tip of the equipment. Both the researcher in charge and the participant wore protective glasses during the laser application, avoiding any contact of the light beam with the eyes.

### Stage 3 - Reassessment

After laser application, individuals underwent the same tongue pressure assessment procedures reported in stage 1.

Measures were taken immediately after irradiation in all groups. Thus, they were reassessed immediately after ending the 4 minutes of irradiation in G4; immediately after ending the 7 minutes of irradiation in G7; and after 4 or 7 minutes of irradiation in PG, as randomly defined for each participant in this group. The interval between measures in CG took 4 minutes, equivalent to the time spent in the 4 J application.

This study was double-blind. The researcher who assessed tongue pressure did not know to which group each participant was allocated. Likewise, participants did not know in which group they were.

### Data analysis

The collected data were recorded in a Microsoft Excel spreadsheet and subsequently analyzed using measures of central tendency and dispersion. The Shapiro-Wilk test showed that the data were normally distributed. The Kruskal-Wallis test verified the homogeneity regarding age and maximum anterior and posterior tongue pressure measured before irradiation. The paired t-test compared pre- and post-intervention pressure, with a 5% significance level and a 95% confidence interval in all analyses.

## RESULTS

The results indicated that the groups were homogeneous regarding sex (p = 0.239), age (p = 0.949), maximum anterior tongue pressure (p = 0.333), and maximum posterior tongue pressure (p = 0.394) before laser application.

Tables 1 and 2 compare the maximum anterior and posterior tongue pressure, respectively, before and after irradiation in each group. There was no significant difference in the pre- and post-irradiation comparisons for any of the study groups.

**Table 1:** Maximum anterior tongue pressure (kPa) before and after laser application

Group	G4		G7		CG		PG	
	Before	After	Before	After	Before	After	Before	After
Mean	45.7	45.9	47.3	47.7	45.4	43.5	54.2	51.1
SD	12.5	13.5	14.6	16.4	14.2	15.5	12.4	14.7
Minimum	39.5	39.2	40.1	39.5	38.3	35.8	48.1	43.7
Maximum	52.0	52.6	54.5	55.9	52.5	51.2	60.4	58.4
p-value	0.921		0.838		0.187		0.056	

Paired t-test. 5% significance level

Caption: G7 = group irradiated with 7 J per point; G4 = group irradiated with 4 J per point; PG = placebo group; CG = control group, SD = standard deviation

**Table 2:** Maximum posterior tongue pressure (kPa) before and after laser application

Group	G4		G7		CG		PG	
	Before	After	Before	After	Before	After	Before	After
Mean	45.9	46.1	47.5	47.6	40.1	38.7	49.1	47.5
SD	14.3	12.2	15.7	15.4	13.5	15.3	13.3	11.2
Minimum	38.8	40.0	39.7	39.9	33.3	31.0	42.5	42.0
Maximum	53.0	52.1	55.4	55.3	46.8	46.3	55.7	53.1
p-value	0.898		0.992		0.371		0.426	

Paired t-test. 5% significance level

Caption: G7 = group irradiated with 7 J per point; G4 = group irradiated with 4 J per point; PG = placebo group; CG = control group, SD = standard deviation

## DISCUSSION

This study found no significant difference between the maximum anterior and posterior tongue pressures before and immediately after irradiation in any irradiated group. However, the mean maximum tongue pressure increased slightly in the irradiated groups and decreased in the non-irradiated groups.

The findings of this study agree with other ones that also found no increase in muscle strength after infrared laser irradiation in skeletal muscles^(20-23)^. A study with healthy older women found that 56 J infrared laser irradiation on the rectus femoris muscle did not increase its strength^(20)^. Other authors evaluated whether irradiation associated with resistance training in healthy women would increase quadriceps performance. They did not find significant changes in respiratory capacity and muscle performance using infrared laser irradiation with a total energy of 18 J^(21)^ but found an increase in resistance to fatigue. Other studies^(22-24)^ found a decrease in electromyographic fatigue after infrared laser irradiation with a total energy of 60 J in the rectus femoris, vastus medialis, and vastus lateralis muscles in healthy men^(22)^, as well as after infrared laser irradiation with a total energy of 25 J on the soleus muscle of healthy women^(23)^. Such studies suggest that photobiomodulation seemingly exerts a greater influence on fatigue than on muscle strength.

Other studies, however, have found an increase in muscle performance after photobiomodulation with infrared laser. Toma et al.^(24)^ found better performance in quadriceps muscle strength gain after irradiation with 56 J in healthy older women. Baroni et al.^(25)^ found greater strength gain and muscle hypertrophy in individuals irradiated with 240 J in the quadriceps. Leal et al.^(26)^ found an increase in the number of repetitions, time before exhaustion, and change in biochemical markers in the biceps in the group irradiated with 60 J of infrared laser in relation to the placebo group. Such contradictory findings in the literature seem to indicate that there are ideal dosimetric activation parameters for each muscle. The lack of difference between before and after irradiation in the present study may be related to its dosimetry, which may not have been enough to produce the expected effects. Another possibility is that the number of irradiated points was not enough to cover the entire muscle. However, performing more points with the equipment used in this research would imply that the participant would have to spend more time with their mouth open, which could fatigue the suprahyoid muscles.

It is also important to question the differences in the behavior of muscle groups when exposed to radiation. Most studies on the effects of light on muscle performance were conducted with striated skeletal muscles, such as the biceps, quadriceps, and rectus femoris^(9)^. Orofacial muscles have particularities, such as smaller dimensions and insertions into other muscles. Therefore, it can be assumed that they have specific behaviors when exposed to radiation and do not necessarily respond to the same parameters applied to other muscles. This research used the 7 J dose based on the study by Mouffron et al.^(15)^, who investigated the immediate effects of photobiomodulation on maximum lip pressure with different amounts of energy. The authors found greater changes in the maximum pressure of the orbicularis oris muscle with 7 J than with the other doses tested (1 J and 4 J)^(15)^. However, the finding cannot be generalized to the tongue muscles, whose fiber structure and composition differ substantially from those of the lip^(27,28)^.

Another factor that may have influenced the findings is the time interval between irradiation and reassessment. Since this is a study of immediate effects, the time between irradiation and measurement may have been insufficient for the light to interact with the tissue to produce the desired effects. In this sense, researchers^(14)^ also found no difference in electromyographic fatigue of the lips before and immediately after irradiation with 4 J at wavelengths of 660 and 808 nm. Experiments in animals indicate better muscle performance results 6 hours after irradiation^(29,30)^. Leal-Junior et al.^(31)^ recommend application immediately before exercise when the objective is to gain strength, but the guidance was based on studies with large muscle groups. The applicability of this guidance to orofacial muscles needs to be further investigated.

The need to open the mouth and slightly protrude the tongue during the irradiation may have also influenced the research findings. This may have caused muscle fatigue in non-irradiated muscles, such as the genioglossus (used in tongue propulsion) and the suprahyoid muscles (used in mandibular lowering). Individuals with signs and symptoms of temporomandibular disorder were not included in the research to minimize the discomfort caused by this position. Moreover, the requested tongue protrusion was slight, keeping it in the intraoral space. However, the application time, especially in the 7 J group (7 minutes), was rather long to maintain this tongue position. Research indicates that tongue pressure on the palate involves contraction of the genioglossus muscle, with a smaller contribution from the suprahyoid muscles^(32)^, especially when the pressure is applied with the IOPI bulb in the anterior region^(33)^. Therefore, further research should consider applying to points in the submental region.

The limitations of this study were the immediate data collection and the lack of longitudinal follow-up. To date, this is the first study to investigate the effects of photobiomodulation on tongue muscle performance. Therefore, it is suggested that future research investigate different therapeutic windows, the association of different wavelengths, and especially the long-term effects of photobiomodulation associated with myofunctional therapy.

## CONCLUSION

No statistically significant differences were found between the maximum anterior and posterior tongue pressures before and after intervention in any of the study groups.
