# Supplementary material for: Immediate effects of photobiomodulation on maximum tongue pressure: a randomized clinical study
Source: Codas. 2025 Feb 21;37(2):e20240139. doi: 10.1590/2317-1782/e20240139en (PMC11864764; doi:10.1590/2317-1782/e20240139en)
Supplement: Translation

# Translation: Efeitos imediatos da fotobiomodulação na pressão máxima da língua: estudo clínico randomizado

**DOI:** 10.1590/2317-1782/e20240139pt

**Authors:** Ester Florens Guerra Gouvêa, Lorena Moreira Marra, Vanessa Mouffron Novaes Alves, Mariana Rodrigues Batista, Andréa Rodrigues Motta, Renata Maria Moreira Moraes Furlan

**Affiliations:** 1 Graduação em Fonoaudiologia, Universidade Federal de Minas Gerais – UFMG - Belo Horizonte (MG), Brasil.; 2 Programa de Pós-graduação em Ciências Fonoaudiológicas, Universidade Federal de Minas Gerais – UFMG - Belo Horizonte (MG), Brasil.; 3 Departamento de Fonoaudiologia, Universidade Federal de Minas Gerais – UFMG - Belo Horizonte (MG), Brasil.

**Keywords:** Terapia com Luz de Baixa Intensidade, Força Muscular, Fonoaudiologia, Sistema Estomatognático, Língua

## Abstract

**Objetivo:**

verificar os efeitos imediatos da fotobiomodulação com laser infravermelho sobre a pressão máxima da língua.

**Método:**

trata-se de um estudo clínico randomizado, realizado com 72 indivíduos adultos, saudáveis, de ambos os sexos, com média de idade de 24,6 anos, desvio-padrão de 4,6, com ausência de anomalia craniofacial, sem sinais e/ou sintomas de disfunção temporomandibular, sem contraindicação à fototerapia e que não faziam uso contínuo de medicamentos miorrelaxantes e/ou anti-inflamatórios. Foram excluídos da amostra os participantes com alterações no frênulo de língua. Foi realizada a medição da pressão máxima da língua utilizando-se o Iowa Oral Performance Instrument (IOPI) antes e após irradiação com laser de baixa intensidade no comprimento de onda de 808 nm, sendo três pontos na porção anterior da língua e três na posterior. Os participantes foram alocados em quatro grupos com 18 indivíduos cada, de forma randomizada: o G4, irradiado com 4 J por ponto; o G7, irradiado com 7 J por ponto; o GC que não recebeu irradiação; e o GP, submetido aos mesmos procedimentos do G4 e G7, mas sem a ativação do laser, ou seja, sem irradiação.

**Resultados:**

não foram constatadas diferenças estatisticamente significantes entre as pressões máximas anteriores e posteriores da língua quando comparados os valores pré e pós-intervenção. Embora sem significância estatística, os grupos que receberam a irradiação apresentaram leve aumento da média, enquanto os grupos não irradiados apresentaram diminuição.

**Conclusão:**

não foram constatadas diferenças entre as pressões máximas anteriores e posteriores da língua, quando comparados os valores de pressão pré e pós-intervenção.

## INTRODUÇÃO

A língua é um órgão muscular pertencente ao sistema estomatognático, que atua em funções relacionadas à nutrição e à comunicação^(1)^. É composta por músculos intrínsecos e extrínsecos, com fibras em diferentes direções (vertical, longitudinal e transversal), o que lhe confere amplas possibilidades de movimentação^(2)^. Os músculos intrínsecos têm origem e inserção na própria língua e são responsáveis pelas suas mudanças de forma, enquanto os músculos extrínsecos têm origem em estruturas adjacentes e atuam, principalmente, na sua movimentação em todas as direções^(3)^.

A pressão da língua pode ser definida como a capacidade de esta estrutura realizar força em uma determinada área^(4)^. Trata-se de uma variável constantemente avaliada na literatura científica, visto que algumas condições clínicas estão associadas à diminuição da força da língua, por exemplo, a respiração oral^(5)^, a apneia obstrutiva do sono^(6)^ e o ronco primário^(6)^. Indivíduos que apresentam alterações dessa força podem desenvolver prejuízos nas funções do sistema estomatognático^(7)^.

Quando detectada a diminuição da força da língua, o processo de reabilitação muscular é, geralmente, composto pela mioterapia e pela terapia miofuncional^(1)^. A mioterapia baseia-se na realização de exercícios musculares, enquanto a terapia miofuncional utiliza o treino funcional assistido para reabilitar as estruturas e as funções do sistema estomatognático^(8)^.

Atualmente, um dos recursos adjuvantes que tem sido utilizado na mioterapia para acelerar o processo de recuperação da força muscular é a fotobiomodulação, em específico, o uso do laser de baixa intensidade^(9)^. O laser tem ação biomoduladora no organismo, ou seja, atua sobre os tecidos estimulando ou inibindo as funções químicas e/ou fisiológicas das células. Dessa forma, a fotobiomodulação pode aprimorar o desempenho muscular, reduzir a fadiga, aumentar a força e propiciar o relaxamento da musculatura a ser trabalhada^(10)^. Tais efeitos justificam-se pelo fato de que a luz age sobre as mitocôndrias das células, especificamente no processo de respiração celular, intensificando a produção de adenosina trifosfato (ATP), que fornece energia para a contração muscular^(11)^. Apesar de comprovada a eficácia da fotobiomodulação para aumento de força em alguns grupos de músculos esqueléticos^(9,12,13)^, poucos são os estudos que envolvem a musculatura orofacial^(14,15)^.

Um estudo, realizado com mulheres com idades entre 19 e 43 anos comparou os efeitos imediatos da fotobiomodulação na fadiga eletromiográfica, utilizando o laser nos comprimentos de onda 660 nanômetros (nm) e 808 nm sobre o músculo orbicular da boca. Foram aplicados 4 J de energia em dois pontos do lábio superior e dois do inferior, totalizando 8 J por segmento. Contudo, não foram encontradas diferenças significativas nas medidas antes e após a irradiação^(14)^. Outro estudo, com uma amostra que contemplava homens e mulheres na faixa etária de 18 a 33 anos, comparou a diferença na pressão dos lábios antes e após a aplicação do laser. Neste caso, foram aplicadas três intensidades diferentes de radiação: 1 J, 4 J e 7 J, em seis pontos do músculo orbicular da boca, sendo observado que a dose de 7 J de energia foi capaz de melhorar o desempenho do músculo orbicular da boca, aumentando a pressão máxima^(15)^.

Não foi identificado, até o momento, nenhum estudo que tenha investigado o efeito da fotobiomodulação sobre a musculatura da língua, portanto, não há evidências científicas que validem a fotobiomodulação para essa musculatura e indiquem qual é a dose mais eficaz. Diante do exposto, o objetivo geral deste estudo foi investigar os efeitos imediatos da fotobiomodulação sobre a pressão de língua, sendo a hipótese inicial de que a irradiação de baixa intensidade em comprimento de onda infravermelho seria capaz de aumentar a pressão máxima da musculatura da língua.

## MÉTODO

Trata-se de um ensaio clínico randomizado, duplo-cego, realizado após a aprovação do Comitê de Ética em Pesquisa da Universidade Federal de Minas Gerais, sob o número de CAAE 17622619.0.0000.5149 e o parecer 3.581.053. Todos os participantes assinaram o Termo de Consentimento Livre e Esclarecido (TCLE). A pesquisa foi registrada na plataforma de ensaios clínicos REBEC sob o número RBR 7p58r6.

O estudo foi realizado no Observatório de Saúde Funcional em Fonoaudiologia da Faculdade de Medicina da UFMG. A amostra, não-probabilística, foi composta por 72 indivíduos adultos, alunos e/ou funcionários da instituição, sendo 54 do sexo feminino e 18 do sexo masculino, com idades entre 19 e 40 anos (média=24,6, desvio padrão=4,6). Foram adotados como critérios de inclusão: ausência de anomalia craniofacial, de sinais e/ou sintomas de disfunção temporomandibular e de contraindicação à fototerapia. Para serem incluídos na pesquisa, os participantes também não podiam fazer uso contínuo de medicamentos miorrelaxantes e/ou anti-inflamatórios. Foram consideradas contraindicações para a fotobiomodulação: gravidez, glaucoma, lesão sem diagnóstico sobre a área a ser irradiada ou próxima a ela, infecção no local da aplicação e histórico de câncer. Foram excluídos da amostra os participantes com alterações no frênulo de língua, diagnosticadas por meio do Protocolo de Avaliação do Frênulo da Língua^(16)^.

Para coleta dos dados foram cumpridas três etapas: avaliação inicial, aplicação do laser de baixa intensidade e reavaliação.

### Etapa 1 - Avaliação inicial

Inicialmente, foi realizada uma entrevista com o participante para averiguação dos critérios de elegibilidade (ausência de anomalia craniofacial, de sinais e/ou sintomas de disfunção temporomandibular, de gravidez, de glaucoma, de lesão na área a ser irradiada ou próxima a ela, de infecção no local da aplicação, de histórico de câncer e de uso contínuo de medicamentos miorrelaxantes e/ou anti-inflamatórios), bem como a avaliação do frênulo lingual, por meio do Protocolo de Avaliação do Frênulo da Língua^(16)^. Na sequência, a avaliação da pressão máxima da língua foi realizada utilizando-se o Iowa Oral Performance Instrument (IOPI). Trata-se de um aparelho constituído por um bulbo de ar ligado a um transdutor de pressão, que permite mensurar a pressão máxima e a resistência muscular. O balão de ar apresenta 3,5 cm de comprimento, 1,0 cm de diâmetro e conecta-se a um tubo plástico de 11,5 cm. À medida em que esse bulbo é pressionado, o aparelho capta a mudança de pressão gerada, fornecendo valores em kPa que podem ser visualizados na tela do próprio equipamento. O IOPI é o instrumento padrão ouro para avaliação objetiva da pressão e resistência da língua^(17)^.

Os participantes foram orientados a permanecerem sentados em uma cadeira, com flexão de 90º entre quadril, joelho e tornozelos e postura ereta, guiada pelo Plano de Frankfurt, durante as medições da pressão da língua. As medições foram feitas posicionando o bulbo, primeiramente, na porção anterior e, em seguida, na porção posterior da língua.

Para a medida de pressão máxima da porção anterior, o bulbo foi posicionado atrás dos incisivos centrais, na parte central da língua. Para mensurar a pressão máxima posterior, por sua vez, o bulbo foi posicionado com o seu limite anterior paralelo ao início dos primeiros molares^(18)^. A mesma instrução foi dada para todos os participantes, que foram solicitados a pressionar o bulbo contra o palato com a língua, com a máxima força possível, por aproximadamente 2 segundos. Foram obtidas três medidas em cada posição (anterior e posterior da língua), respeitando um intervalo de 30 segundos entre elas^(18)^. Foi considerada para análise a média entre os três valores de pressão obtidos em cada posição.

### Etapa 2: Aplicação do laser de baixa intensidade

A fotobiomodulação foi realizada com laser de baixa intensidade, utilizando o equipamento da marca DMC®, modelo Therapy EC (São Paulo, Brasil). Os parâmetros de irradiação utilizados estão descritos no Quadro 1.

**Quadro 1:** Parâmetros da fotobiomodulação

Parâmetros de irradiação	Valores nos grupos G4 e G7
Comprimento de onda	808 nm (infravermelho)
Modo de operação	Contínuo
Potência de saída	100 mW
Diâmetro do spot de saída	3,54 mm
Área do spot de saída	0,09842 cm^2^
Energia por ponto	4 J e 7 J
Densidade de energia (fluência) por ponto	40,64 J/cm^2^ (G4) e 71,12 J/cm^2^ (G7)
Tempo de aplicação por ponto	40 s (G4) e 70 s (G7)
Número de pontos	6
Tempo de total de irradiação	4 min (G4) e 7 min (G7)
Energia total	24 J (G4) e 42 J (G7)
Modo de aplicação	Modo estacionário com contato

Os 72 participantes da pesquisa foram alocados de forma aleatória por sorteio em quatro grupos com 18 participantes cada um. A descrição detalhada de cada grupo e a energia irradiada estão descritas a seguir:

- Grupo 1 (G4): composto por 14 participantes do sexo feminino e quatro do sexo masculino, com média de idade de 25,5 anos, DP de 5,9, mínimo de 20 e máximo de 36 anos. Os indivíduos foram irradiados com 4 J de energia por ponto, sendo a energia total de 24 J e o tempo total de irradiação de quatro minutos;
- Grupo 2 (G7): composto por 14 participantes do sexo feminino e quatro do sexo masculino, com média de idade de 24,6 anos, DP de 4,0, mínimo de 20 e máximo de 33 anos. Irradiação com 7 J de energia por ponto, sendo a dose total de 42 J e o tempo total de irradiação de sete minutos;
- Grupo Controle (GC): composto por 15 participantes do sexo feminino e três do sexo masculino, com média de idade de 24,6, DP de 5,0, mínimo de 19 e máximo de 40 anos. Os participantes não foram irradiados.
  ● Grupo Placebo (GP): composto por dez participantes do sexo feminino e oito do sexo masculino, com média de idade de 23,8, DP de 3,6, mínimo de 19 e máximo de 31 anos. Os participantes foram submetidos aos mesmos procedimentos do G4 e G7, respeitando-se o mesmo tempo de aplicação e o mesmo tempo total de irradiação (quatro ou sete minutos, definido de forma aleatória para cada participante), mas o aparelho não foi acionado.

Em todos os grupos, a irradiação foi realizada de modo pontual, com contato, em seis pontos sobre a língua, sendo três pontos no terço anterior e três pontos na porção posterior, com distância de 1 cm entre eles, tal qual representado na Figura 1^(19)^.

**Figura 1:** Pontos de aplicação do laser na superfície da língua

Fonte: Pexels^(19)^ (Adaptado pelo autor)

Houve a necessidade de evitar que o procedimento da irradiação provocasse a fadiga da musculatura lingual. Primeiramente, foram irradiados, de forma ininterrupta, os pontos localizados na porção anterior da língua, seguida da posterior. Além disso, durante a fotobiomodulação, os participantes foram orientados a abrir a boca e manter a língua no assoalho de forma confortável, conforme mostrado na Figura 2.

**Figura 2:** Posicionamento de aplicação do laser

Fonte: Acervo do autor (2023)

A irradiação foi feita encostando a ponteira do equipamento sobre a mucosa da língua do participante. O aparelho foi higienizado com álcool 70% antes de cada aplicação e, também, utilizou-se um plástico filme descartável para encapar a ponteira do equipamento. Tanto o pesquisador responsável quanto o participante utilizaram óculos de proteção durante a aplicação do laser, evitando qualquer contato do feixe de luz com os olhos.

### Etapa 3 - Reavaliação

Após a aplicação do laser, os indivíduos foram submetidos aos mesmos procedimentos de avaliação da pressão da língua relatados na Etapa 1.

A medição foi realizada imediatamente após a irradiação, para todos os grupos. Dessa forma, para o G4 a reavaliação foi realizada imediatamente após finalizados os quatro minutos de irradiação; para o G7 imediatamente após os sete minutos de irradiação e, para o GP, após quatro ou sete minutos da irradiação, o que foi definido aleatoriamente para cada participante desse grupo. No grupo controle, o intervalo entre as medições foi de quatro minutos, equivalente ao tempo gasto na aplicação de 4 J.

Esta pesquisa teve caráter duplo-cego. O pesquisador que realizou as avaliações da pressão da língua não sabia em qual grupo cada participante estava alocado. Os participantes também não sabiam em qual grupo se encontravam.

### Análise dos dados

Os dados coletados foram registrados em uma planilha do Microsoft Excel e, posteriormente, analisados por meio de medidas de tendência central e dispersão. Por meio do teste Shapiro-Wilk verificou-se que os dados apresentaram distribuição normal. A homogeneidade em relação à idade e à pressão máxima anterior e posterior da língua mensuradas antes da irradiação foi verificada por meio do teste Kruskal-Wallis. O teste T pareado foi utilizado para as comparações da pressão pré e pós-intervenção, sendo adotado nível de significância de 5% e intervalo de confiança de 95% em todas as análises.

## RESULTADOS

Os resultados indicaram que os grupos são homogêneos em relação a sexo (p=0,239), idade (p=0,949), pressão máxima anterior da língua (p=0,333) e pressão máxima posterior da língua (p=0,394) antes da aplicação do laser.

As Tabelas 1 e 2 apresentam a comparação da pressão máxima anterior e posterior da língua, respectivamente, antes e após a irradiação em cada grupo. Não houve diferença significativa nas comparações pré e pós irradiação para nenhum dos grupos pesquisados.

**Tabela 1:** Pressão anterior máxima da língua (kPa) antes e após a aplicação do laser

Grupo	G4		G7		GC		GP	
	Pré	Pós	Pré	Pós	Pré	Pós	Pré	Pós
Média	45,7	45,9	47,3	47,7	45,4	43,5	54,2	51,1
DP	12,5	13,5	14,6	16,4	14,2	15,5	12,4	14,7
Mínimo	39,5	39,2	40,1	39,5	38,3	35,8	48,1	43,7
Máximo	52,0	52,6	54,5	55,9	52,5	51,2	60,4	58,4
Valor de p	0,921		0,838		0,187		0,056	

Teste T pareado. Nível de significância de 5%

Legenda: G7 = grupo irradiado com 7 J por ponto; G4 = grupo irradiado com 4 J por ponto; GP = grupo placebo; GC = grupo controle, DP = desvio padrão

**Tabela 2:** Pressão posterior máxima da língua (kPa) antes e após a aplicação do laser

Grupo	G4		G7		GC		GP	
	Pré	Pós	Pré	Pós	Pré	Pós	Pré	Pós
Média	45,9	46,1	47,5	47,6	40,1	38,7	49,1	47,5
DP	14,3	12,2	15,7	15,4	13,5	15,3	13,3	11,2
Mínimo	38,8	40,0	39,7	39,9	33,3	31,0	42,5	42,0
Máximo	53,0	52,1	55,4	55,3	46,8	46,3	55,7	53,1
Valor de p	0,898		0,992		0,371		0,426	

Teste T pareado. Nível de significância de 5%

Legenda: G7 = grupo irradiado com 7 J por ponto; G4 = grupo irradiado com 4 J por ponto; GP = grupo placebo; GC = grupo controle, DP = desvio padrão

## DISCUSSÃO

Na presente pesquisa verificou-se ausência de diferença significativa entres as pressões máximas anterior e posterior da língua antes e imediatamente após a irradiação para todos os grupos irradiados. Entretanto, nota-se que os grupos que receberam a irradiação apresentaram leve aumento na média de pressão máxima da língua, enquanto os grupos não irradiados apresentaram redução dessa medida.

Os achados da presente pesquisa concordam com os de outros estudos que também verificaram ausência de aumento da força muscular após irradiação com laser infravermelho em músculos esqueléticos^(20-23)^. Um estudo desenvolvido com mulheres idosas saudáveis verificou que a irradiação com laser infravermelho e energia de 56 J sobre o músculo reto femoral não foi capaz de promover aumento de força desse músculo^(20)^. Outros autores que avaliaram se a irradiação associada ao treino de resistência em mulheres saudáveis seria capaz de aumentar o desempenho muscular em quadríceps não encontraram mudanças significativas na capacidade respiratória e no desempenho da musculatura utilizando irradiação com laser infravermelho e energia de 18 J no total^(21)^, mas verificaram aumento da resistência à fadiga. Outros estudos^(22,23)^ verificaram diminuição da fadiga eletromiográfica, após irradiação com laser infravermelho, energia total de 60 J, nos músculos reto-femoral, vasto medial e vasto lateral em homens saudáveis^(22)^, bem como após irradiação com laser infravermelho, energia total de 25 J, sobre o músculo sóleo de mulheres saudáveis^(23)^. Tais estudos sugerem que a fotobiomodulação parece exercer maior influência sobre a fadiga do que sobre a força muscular.

Outras pesquisas, porém, verificaram aumento no desempenho muscular após a fotobiomodulação com laser infravermelho. Toma et al.^(24)^ encontraram melhor desempenho no ganho de força do músculo quadríceps após irradiação com 56 J em mulheres idosas saudáveis. Baroni et al.^(25)^ verificaram maior ganho de força e hipertrofia muscular em indivíduos irradiados com 240 J em quadríceps. Leal et al.^(26)^ constataram, para o músculo bíceps, aumento do número de repetições, do tempo antes da exaustão e mudança nos marcadores bioquímicos no grupo irradiado com 60 J do laser infravermelho em relação ao grupo placebo. Tais achados contraditórios, presentes na literatura, parecem indicar que existem parâmetros dosimétricos ideais de ativação para cada musculatura. Nesse sentido, a ausência de diferença entre os momentos pré e pós irradiação, verificada na presente pesquisa, pode estar relacionada com a dosimetria selecionada, a qual pode não ter sido suficiente para resultar nos efeitos esperados. Outra possibilidade é que a quantidade de pontos irradiados não tenha sido suficiente para cobrir toda a musculatura. Porém, a realização de mais pontos, com o equipamento utilizado nesta pesquisa, implicaria em maior tempo de permanência do participante com a boca aberta, o que poderia fadigar a musculatura supra-hioidea.

É importante questionar, também, as diferenças dos comportamentos dos grupos musculares em contato com a radiação. A maioria dos estudos sobre os efeitos da luz no desempenho muscular foi realizada com músculos estriados esqueléticos corporais, como bíceps, quadríceps, reto femoral^(9)^. Os músculos orofaciais apresentam particularidades, como dimensões reduzidas e inserções em outros músculos. Sendo assim, pode se presumir que apresentam comportamentos específicos frente à irradiação e que não necessariamente respondem aos mesmos parâmetros aplicados a outras musculaturas. A dose de 7 J foi selecionada para esta pesquisa a partir do estudo de Mouffron et al.^(15)^, que pesquisou os efeitos imediatos da fotobiomodulação sobre a pressão máxima dos lábios para diferentes quantidades de energia. Os autores verificaram que a dose de 7 J promoveu mudanças na pressão máxima do músculo orbicular da boca, superiores às outras doses testadas (1 J e 4 J)^(15)^. Ainda assim, não se pode generalizar o achado para a musculatura da língua, cuja estrutura muscular e composição de fibras musculares difere-se substancialmente das do lábio^(27,28)^.

Outro fator que pode ter influenciado os achados refere-se ao espaço de tempo entre a irradiação e a reavaliação. Acredita-se que, por ser uma pesquisa de efeitos imediatos, o tempo entre a irradiação e a medição pode ter sido insuficiente para a luz interagir com o tecido para produção dos efeitos desejados. Nesse sentido, pesquisadores^(14)^ também verificaram ausência de diferença na fadiga eletromiográfica dos lábios antes e imediatamente após a irradiação com 4 J nos comprimentos de 660 e 808 nm. Experimentos em animais indicam melhores resultados de desempenho muscular após seis horas da irradiação^(29,30)^. Leal-Junior et al^(31)^ orientam a aplicação imediatamente antes do exercício quando o objetivo é o ganho de força, mas a orientação foi feita com base em estudos com grandes grupos musculares. A aplicabilidade dessa orientação para os músculos orofaciais precisa ser melhor investigada.

Pode-se pensar que outro influenciador dos achados da pesquisa foi a necessidade de se realizar a abertura de boca e uma leve protrusão da língua durante o procedimento da irradiação, o que pode ter ocasionado fadiga muscular em músculos não irradiados, como o genioglosso (envolvido na propulsão da língua) e os supra-hioideos (que atuam no abaixamento mandibular). Na tentativa de minimizar o incômodo ocasionado por essa posição, não foram incluídos indivíduos com sinais e sintomas de disfunção temporomandibular na pesquisa e a protrusão da língua solicitada foi leve, mantendo-a no espaço intraoral. Porém, o tempo de aplicação, especialmente para o grupo de 7 J (7 minutos) era consideravelmente aumentado para se manter essa posição da língua. Pesquisas apontam que a pressão de língua no palato envolve a contração do músculo genioglosso, com contribuição, porém menor, dos músculos supra-hioideos^(32)^, especialmente quando a pressão é realizada com o bulbo do IOPI em região anterior^(33)^. Sendo assim, sugere-se que outras pesquisas considerem a inclusão de pontos de aplicação em região submentual.

Constituíram limitações da presente pesquisa o caráter imediato da coleta e a ausência de acompanhamento longitudinal. Até o presente momento, este é o primeiro estudo que investigou os efeitos da fotobiomodulação no desempenho muscular da língua. Dessa forma, abrem-se sugestões para futuras pesquisas que incluem a investigação de diferentes janelas terapêuticas, associação de diferentes comprimentos de onda e, principalmente, pesquisas de efeitos de longo prazo da fotobiomodulação associada à terapia miofuncional.

## CONCLUSÃO

Não foram encontradas diferenças estatisticamente significantes entre as pressões máximas anterior e posterior da língua, na comparação entre os momentos pré e pós-intervenção, em nenhum dos grupos estudados.
